# Supplementary material for: Parents’ awareness, knowledge, and experiences of play and its benefits in child development: A systematic review protocol
Source: PLoS One. 2022 Sep 9;17(9):e0274238. doi: 10.1371/journal.pone.0274238 (PMC9462721; doi:10.1371/journal.pone.0274238)
Supplement: S2 File — (DOCX) [file pone.0274238.s002.docx]

S2 Additional file

Parents’ awareness, knowledge, and experiences of play and its benefits in child development: a systematic review protocol

**Search Strategy**

Interface: EBSCOhost Research

Databases Search Screen: Advanced Search

Database: MEDLINE

Year: 1946 to present

| **Search No** | **Query** | **Expanders** | **Limiters/Expanders** | **Search mode** |
| --- | --- | --- | --- | --- |
| S1 | Play* | Apply related words | English Language  Human  Scholarly (Peer Reviewed) Journals | Find all my search terms |
| S2 | Gam* NOT gamma NOT gambling | Apply related words | English Language  Human  Scholarly (Peer Reviewed) Journals | Find all my search terms |
| S3 | Sport* | Apply related words | English Language  Human  Scholarly (Peer Reviewed) Journals | Find all my search terms |
| S4 | Parent* | Apply related words | English Language  Human  Scholarly (Peer Reviewed) Journals | Find all my search terms |
| S5 | Mother* | Apply related words | English Language  Human  Scholarly (Peer Reviewed) Journals | Find all my search terms |
| S6 | Father* | Apply related words | English Language  Human  Scholarly (Peer Reviewed) Journals | Find all my search terms |
| S7 | Importan* | Apply related words | English Language  Human  Scholarly (Peer Reviewed) Journals | Find all my search terms |
| S8 | Comprehen* | Apply related words | English Language  Human  Scholarly (Peer Reviewed) Journals | Find all my search terms |
| S9 | Perce* | Apply related words | English Language  Human  Scholarly (Peer Reviewed) Journals | Find all my search terms |
| S10 | Attitud* | Apply related words | English Language  Human  Scholarly (Peer Reviewed) Journals | Find all my search terms |
| S11 | Understand* | Apply related words | English Language  Human  Scholarly (Peer Reviewed) Journals | Find all my search terms |
| S12 | Aware* | Apply related words | English Language  Human  Scholarly (Peer Reviewed) Journals | Find all my search terms |
| S13 | Experien* | Apply related words | English Language  Human  Scholarly (Peer Reviewed) Journals | Find all my search terms |
| S14 | Involve* | Apply related words | English Language  Human  Scholarly (Peer Reviewed) Journals | Find all my search terms |
| S15 | Competen* | Apply related words | English Language  Human  Scholarly (Peer Reviewed) Journals | Find all my search terms |
| S16 | Capabil* | Apply related words | English Language  Human  Scholarly (Peer Reviewed) Journals | Find all my search terms |
| S17 | Skill* | Apply related words | English Language  Human  Scholarly (Peer Reviewed) Journals | Find all my search terms |
| S18 | Perspective* | Apply related words | English Language  Human  Scholarly (Peer Reviewed) Journals | Find all my search terms |
| S19 | S1 OR S2 OR S3 |  |  | Find all my search terms |
| S20 | S4 OR S5 OR S6 |  |  | Find all my search terms |
| S21 | S7 OR S8 OR S9 OR S10 OR S11 OR S12 OR S13 OR S14 OR S15 OR S16 OR S17 OR S18 |  |  | Find all my search terms |
| S22 | S19 AND S20 AND S21 |  |  | Find all my search terms |
