# Supplementary material for: Parents’ awareness, knowledge, and experiences of play and its benefits in child development: A systematic review protocol
Source: PLoS One. 2022 Sep 9;17(9):e0274238. doi: 10.1371/journal.pone.0274238 (PMC9462721; doi:10.1371/journal.pone.0274238)
Supplement: S3 File — (DOCX) [file pone.0274238.s003.docx]

S3 Additional file

Parents’ awareness, knowledge, and experiences of play and its benefits in child development: a systematic review protocol

**Data extraction form for quantitative studies**

| **Administration Information** | | | |
| --- | --- | --- | --- |
| Title |  | | |
| Authors |  | | |
| Year |  | | |
| Journal |  | | |
| Country of origin |  | | |
| **Study Information** | | | |
|  | Description | Summary | |
| Objectives | Specific objectives mentioned in the study |  | |
| Study design | Study design as identified in the paper. |  | |
| Setting | Setting, locations, and relevant dates, including periods of recruitment, exposure, follow-up, and data collection |  | |
| Eligibility criteria | Inclusion and exclusion criteria |  | |
|  | Sources and methods of selection of participants |  | |
| Variables | List all outcomes, exposures, predictors, potential confounders, and effect modifiers. |  | |
|  | Definitions used |  | |
| Data sources/ measurement | Tools used to measure variables of interest, their psychometric properties |  | |
| Bias | Any efforts to address potential sources of bias |  | |
| Study size | How the study size was arrived at |  | |
| Statistical methods | List all statistical methods used |  | |
| Participants | Numbers of individuals at each stage of study—eg numbers potentially eligible, examined for eligibility, confirmed eligible, included in the study, completing follow-up, and analysed |  |  |
|  | Reasons for non-participation at each stage |  |  |
| Descriptive data | Characteristics of study participants (eg demographic, clinical, social) |  |  |
|  | Number of participants with missing data for each variable of interest |  |  |
| Outcome data | Summary of measures over time and time points |  |  |
| Main results | Percentages, odds ratio, p values, other results |  |  |
| Other analyses | Report other analyses done—eg analyses of subgroups and interactions, and sensitivity analyses |  |  |
| Summary of findings | Summarise key findings |  |  |
| Funding | Give the source of funding and the role of the funders for the present study and, if applicable, for the original study on which the present article is based |  |  |

**Data extraction form for qualitative studies**

| **Administration Information** | |
| --- | --- |
| Title |  |
| Authors |  |
| Year |  |
| Journal |  |
| Country of origin |  |
| **Study Information** | |

| Personal characteristics of the researcher | Which author/s conducted the interview or focus group? |  |
| --- | --- | --- |
|  | What were the researcher's credentials? E.g. PhD, MD |  |
|  | What was their occupation at the time of the study? |  |
|  | Was the researcher male or female? |  |
|  | What experience or training did the researcher have? |  |
| Relationship with participants | Was a relationship established prior to study commencement? |  |
|  | What did the participants know about the researcher? e.g. personal goals, reasons for doing the research |  |
|  | What characteristics were reported about the interviewer/facilitator? e.g. Bias, assumptions, reasons and interests in the research topic |  |
| Theoretical framework | What methodological orientation was stated to underpin the study? e.g. grounded theory, discourse analysis, ethnography, phenomenology, content analysis |  |
| Participant selection | How were participants selected? e.g. purposive, convenience, consecutive, snowball |  |
|  | How were participants approached? e.g. face-to-face, telephone, mail, email |  |
|  | How many participants were in the study? |  |
|  | How many people refused to participate or dropped out? Reasons? |  |
| Setting | Where was the data collected? e.g. home, clinic, workplace |  |
|  | Was anyone else present besides the participants and researchers? |  |
|  | What are the important characteristics of the sample? e.g. demographic data, date |  |
| Data collection | Were questions, prompts, guides provided by the authors? Was it pilot tested? |  |
|  | Were repeat interviews carried out? If yes, how many? |  |
|  | Did the research use audio or visual recording to collect the data? |  |
|  | Were field notes made during and/or after the interview or focus group? |  |
|  | What was the duration of the interviews or focus group? |  |
|  | Was data saturation achieved? |  |
|  | Were transcripts returned to participants for comment and/or correction? |  |
| Data analysis | How many data coders coded the data? |  |
|  | Were themes identified in advance or derived from the data? |  |
|  | What software, if applicable, was used to manage the data? |  |
|  | Process by which themes/inferences were derived |  |
|  | Did participants provide feedback on the findings? |  |
| Results | Central and sub-themes with brief description |  |
|  | Copy important participant quotes |  |
|  | Author’s key conclusions |  |
|  | Transferability |  |
|  | Limitations |  |
| Funding |  |  |
